# Monitoring multiple myeloma by idiotype-specific peptide binders of tumor-derived exosomes

**DOI:** 10.1186/s12943-017-0730-8

**Published:** 2017-10-13

**Authors:** Enrico Iaccino, Selena Mimmi, Vincenzo Dattilo, Fabiola Marino, Patrizio Candeloro, Antonio Di Loria, Danilo Marimpietri, Antonio Pisano, Francesco Albano, Eleonora Vecchio, Simona Ceglia, Gaetanina Golino, Antonio Lupia, Giuseppe Fiume, Ileana Quinto, Giuseppe Scala

**Affiliations:** 1 Department of Experimental and Clinical Medicine, University of Catanzaro “Magna Graecia,”, Catanzaro, Italy; 2 Stem Cell and Cellular Therapy Laboratory, G. Gaslini Institute, Genoa, Italy

## Abstract

Tumor-derived exosomes (TDEs) play a pivotal role in tumor establishment and progression, and are emerging biomarkers for tumor diagnosis in personalized medicine. To date, there is a lack of efficient technology platforms for exosome isolation and characterization. Multiple myeloma (MM) is an incurable B-cell malignancy due to the rapid development of drug-resistance. MM-released exosomes express the immunoglobulin B-cell receptor (Ig-BCR) of the tumor B-cells, which can be targeted by Idiotype-binding peptides (Id-peptides). In this study, we analyzed the production of MM-released exosomes in the murine 5T33MM multiple myeloma model as biomarkers of tumor growth. To this end, we selected Id-peptides by screening a phage display library using as bait the Ig-BCR expressed by 5T33MM cells. By FACS, the FITC-conjugated Id-peptides detected the MM-released exosomes in the serum of 5T33MM-engrafted mice, levels of which are correlated with tumor progression at an earlier time point compared to serum paraprotein. These results indicate that Id-peptide-based recognition of MM-released exosomes may represent a very sensitive diagnostic approach for clinical evaluation of disease progression.

**Electronic supplementary material:**

The online version of this article (10.1186/s12943-017-0730-8) contains supplementary material, which is available to authorized users.

## Background

Multiple myeloma (MM) is a clonal B-cell malignancy accounting for more than 10% of hematologic cancers, and is characterized by the aberrant expansion of bone marrow plasma cells releasing a high level of monoclonal immunoglobulin (mIg) in the blood, so called paraprotein [1]. MM remains largely incurable due to the rapid development of aggressive, drug-resistant phenotypes [2]. Monitoring MM progression is a crucial step for determining the stage of disease and choosing the most appropriate therapy. In this context, there is an urgent need to develop novel diagnostic approaches allowing the non-invasive early detection of tumor growth and the efficient monitoring of tumor progression [3].

First identified in the mid-80s [4] and initially classified as unfunctional “garbage bags” containing unwanted cellular constituents, exosomes represent a promising tool for novel diagnostic options in the diagnosis of malignant diseases [5]. Indeed, recent evidence demonstrated the utility of microvesicles in detecting relapse weeks before existing clinical tests, highlighting the sensitivity and capacity for microvesicles in monitoring disease progression and minimal residual disease in myeloma patients [6]. Exosomes are vesicles of 30–130 nm in diameter released by different cell types and detectable in all biological fluids [7] and supernatants of cultured cells [8]. Exosomes contain a wide range of RNA and proteins, playing an important role in cell-to-cell communication [9]. In particular, exosomes are involved in the regulation of the immune response, antigen presentation [10], tumor survival [11], cell migration [12], tumor invasion [13], cell differentiation and angiogenesis [14]. Reflecting the genomic and proteomic profile of their parental cells, circulating serum exosomes are potential biomarkers in predicting cancer burden with relevant impact for personalized therapy [15]. Although several methods have been developed for exosome purification, none of them clearly distinguish between normal and tumor-derived exosomes (TDEs), or avoid contamination by shed membrane vescicles [16]. Even if the mechanism of expression remains not completely defined, it is worthwhile that MM-released exosomes constitutively express on their surface the immunoglobulin of B-cell receptor (Ig-BCR) derived from the parental tumor B-cell, and thus they can be reliable tumor markers [17, 18].

In the last few years, we successfully validated the screening of random peptide libraries (RPLs) as a method to identify peptides binders of soluble immunoglobulins (Igs) [19] transmembrane receptors [20, 21] and biomaterials [22]. In particular, we identified peptide binders of the Ig-BCR idiotypic determinants (hereafter named “Id-peptides”) that are expressed on the surface of the A20 murine B-cell lymphoma, which revealed to be sensitive tools for in vivo tumor detection and tumor-specific delivery of radionuclides, fluorophores, siRNAs and nanoparticles [23].

In this study, we addressed the question whether MM-released exosomes detected by Id-peptides could allow a more efficient monitoring of tumor growth compared to the standard paraprotein assay. To this end, we measured the tumor growth and serum MM-released exosomes in vivo in the 5T33MM murine model [24]. 5T33MM-engrafted mice develop a highly aggressive MM form, presenting biological and genetic characteristics similar to the human disease, and thus it represents one of the most reliable MM preclinical model [25].

## Methods

### Cell lines and immunoglobulin purification

5T33MM, A20 and IM9 B cell lines bear surface Igs that are secreted in the culture medium. Cells were grown in RPMI medium, supplemented with 10% fetal bovine serum, 50 units/ml penicillin, 50 μg/ml streptomycin and 2 mM L-glutamine.

B-cells from MM patient and healthy donor were isolated by negative selection from whole blood using RosetteSep Human B Cell Enrichment Cocktail [Stem Cell Technology, Vancouver, Canada]*,* as previously described [26].

Igs were purified from the culture supernatants by using the Mab Trap™ antibody purification Kit [GE Healthcare, Little Chalfont, UK], according to the manufacturer’s instructions.

### Selection and amplification of phage ligands of 5T33MM Ig

The Ph.D.-C7C Phage Display Peptide Library kit was purchased from New England Biolabs [NEB, Ipswich, Massachusetts, US]. The screening of phage displayed library was performed using the bait 5T33MM Igs, as previously reported [22]. Briefly, the streptavidin-conjugated beads [Thermo Fisher, Waltham, Massachusetts, US] were coated with 5T33MM Igs and incubated with 1 × 10^11^ phages overnight at 4 °C. Beads were extensively washed with PBS supplemented with 0.05% Tween-20 to remove unbound phage. 5T33MM Igs-interacting phages were eluted with 0.2 M glycine-HCl (pH 2.2, 1 mg/mL BSA) followed by the addition of neutralizing solution (1 M Tris-HCl pH 9.1). Ultimately, 3 cycles of panning were performed. Plaques of lysis from isolated phages were transferred to nitrocellulose filters, and membranes were blocked with PBS 1X, 0.1% NP-40; 5% milk; 0.02% NaN3 and then incubated for 2 h at RT with 100 μg of purified 5T33MM Igs. After washing, membranes were hybridized with an alkaline phosphatase-conjugated anti-mouse IgG [Sigma Aldrich, Saint Louis, Missouri, US] (dilution 1:5000) and then washed 6 times. Immunoreactive phage clones were detected by BCIP/NBT premixed substrate [Thermo Fisher].

### Synthesis and in vitro evaluation of id-peptides binding to the 5T33MM Ig

Single strand DNA (ssDNA) was extracted from the selected phage clones by phenol/chlorophorm purification followed by ethanol precipitation. DNA fragment codifying the peptide ligand was amplified by PCR and sequenced to get the primary structure of amino acid sequence for the peptide synthesis. Synthesized peptides were purchased from Caslo Laboratory ApS [Caslo, Kongens Lyngby, DE]. The specific binding of peptides to the 5T33MM sIgG was analyzed by ELISA, as follows. Streptavidin coated 96 well plates were washed extensively and supplemented with biotin-conjugated peptides by 1 h-incubation at 37 °C; then, after washing and blocking with blocking solution (1X PBS, 0.05% Tween-20, 5% milk), aliquots of 5T33MM sIgG (1 μg/ml in blocking buffer) were added overnight at 4 °C. Wells were extensively washed and coated with the anti-mouse IgG (Fc-specific) alkaline phosphatase-conjugated [Sigma Aldrich] for 1 h at 37 °C, incubated with the alkaline phosphatase substrate [Sigma Aldrich], and analyzed by an ELISA reader at 405 nm [Labsystems multiscan MS].

### In vitro cell binding

Cultured 5T33MM, A20 and IM9 cells as well as primary B cells from MM patient and healthy donor were washed and suspended at the density of 10^6^ cells/ml in FACs Flow Buffer [BD Biosciences]. All subsequent washing and incubations were carried out in the same buffer on ice. Cells were incubated with 20 μg/ml of FITC-conjugated peptides for 10 min on ice, then washed twice, and analyzed by flow cytometry [FACSCalibur, BD Biosciences]. For peptide co-localization with the BCR complex, 5T33MM cells (10^6^ cells/ml) were stained with FITC-conjugated peptides [10 μg/ml) and goat anti-mouse IgG-Alexa fluor 568 [Thermo Fisher]. After extensive washing, cells were mounted under cover slip, and visualized by confocal microscopy [Leica TC-SP2].

### Animal studies

Experiments were conducted in C57BL/KaLwRij mice (8 to 10 weeks-old females) in accordance with the National Institutes of Health Guide for the Care and Use of Laboratory Animals. A total number of 10 mice per group were respectively injected with 10^6^ cells of 5T33MM multiple myeloma cells or A20 B-lymphoma cells. Whole blood samples (250 μL) were collected at baseline and then weekly after tumor inoculation by retro-orbital bleeding.

### Isolation of MM-released exosomes

Exosomes were isolated from 250 μL of blood and 10 mL of cell supernatant respectively using ExoQuick solutions [System Biosciences – SBI, Palo Alto, California, US] and ExoQuick-TC™ [System Biosciences], according to the manufacturer’s protocol. Briefly, serum and cell supernatants were centrifuged at 3000×*g* for 15 min to remove cells and cell debris. The supernatant was transferred to a sterile vessel and the recommended volume of ExoQuick solution or ExoQuick-TC™ were added to the bio-fluids. After brief vortex, samples were stored 30 min at 4 °C and then centrifuged at 1500 x *g* for 30 min at room temperature. After removing the supernatant, the pellet was suspended in nuclease-free water.

### Detection of MM-released paraprotein

The levels of serum IgG2b from the blood (250 μL) of tumor-engrafted mice or control mice were measured using the Easy-Titer™ Mouse IgG Assay Kit [Thermo Fisher] according to manufacturer’s instructions.

### Physical characterization of exosomes

The exosomes sample (10 μl) was spread, evaporated by using a vacuum concentrator at 30 °C, and analyzed by scanning electron microscopy [ESEM Quanta 400 instrument; FEI]. Dynamic light scattering and zeta potential determinations were performed with a Nano ZS 90 [Malvern Instruments], allowing the analysis of particles within the range of 1 nm up to 3 μm. For Western blot analysis, the exosomes were lysed in reducing sample buffer [0.25 M Tris–HCl (pH 6.8), 40% glycerol, 8% SDS, 5% 2-mercaptoethanol and 0.04% bromophenol blue] and boiled for 10 min at 95 °C. Proteins were resolved by SDS-PAGE (SDS-polyacrylamide gel electrophoresis), transferred to poly-vinylidene fluoride membranes, blocked with 5% non-fat powdered milk in PBS-T (0.5% Tween-20) and probed with anti-mouse CD63, anti- mouse CD81, or anti-mouse IgG antibodies. Protein bands were detected using X-ray film and enhanced chemiluminescence reagent [GE Healthcare].

### Exosome immunocapture and flow cytometry

Isolated exosomes from serum or cell supernatants were suspended in 1 ml of 1X PBS. Exosome aliquots (500 μl) were labeled with 50 μl of 10X Exo-Red [SBI] according to manufacturer’s instructions. The exosomes were re-isolated using the addition of 100 μl ExoQuick followed by precipitation for 30 min at +4 °C. The labeled exosome pellet was suspended in 500 μl 1X PBS and stained with CD63-coupled magnetic beads provided by SBI’s Exo-Flow IP kit [SBI] and with the FITC-conjugated peptides.

## Results and discussion

**Fig. 1:**
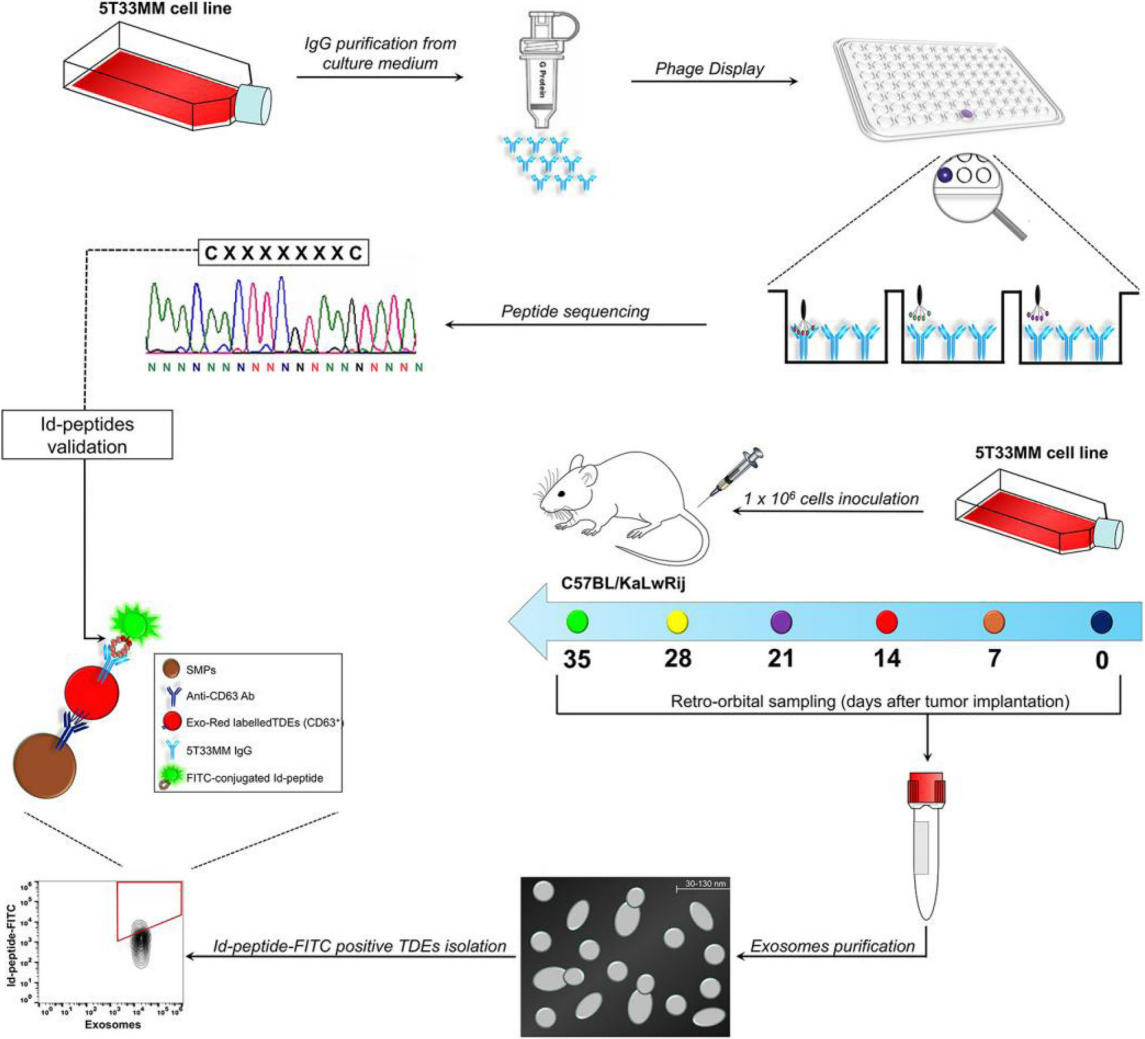
Workflow of the experimental design. The Igs secreted by 5T33MM cells were purified from cell supernatant using Protein G affinity chromatography, and used as bait to isolate phage ligands from the C7C phage-displayed peptide library fused to the M13 minor coat protein. ELISA was performed to select ligands with distinct affinities for their cognate Ig-BCR. Synthetic peptides corresponding to the peptide insert of phage clones were assayed for their antigenic properties out of the phage context. The purified 5T33MM-released exosomes were characterized by scanning electron microscopy (SEM), Zetasizer and Western blotting analysis. SMNPs were decorated with biotinylated anti-CD63, incubated with RED-EXO-labeled exosomes, and analyzed for the binding of FITC-conjugated Id-peptides by flow cytometry. For in vivo analysis, 5T33MM cells (1 × 10^6^) were intravenously injected in C57BL/KaLwRij mice (10 females at 8 weeks of age). Tumor-derived exosomes and paraprotein levels in peripheral blood were monitored every 7 days up to 35 days post-inoculation

**Fig. 2:**
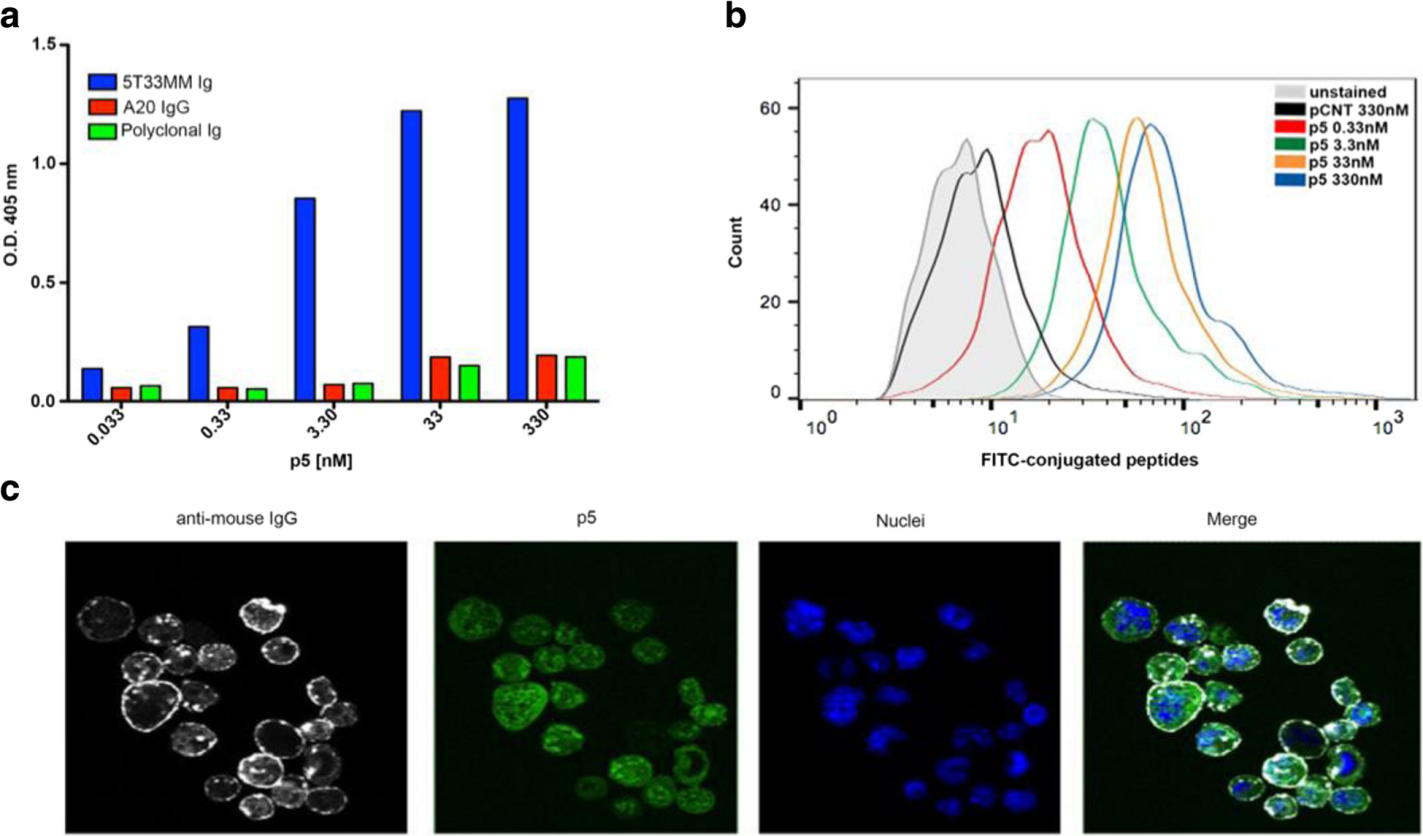
In vitro binding of selected Id-peptides to the 5T33MM-Ig. **a** Concentration-dependent binding of N-biotinylated synthetic Id-peptides to purified 5T33MM-Ig as determined by ELISA. Peptide binding to A20 secreted Igs and polyclonal mouse Igs were included as controls. **b** Concentration-dependent binding of FITC-conjugated p5 peptide and relative control peptide to 5T33MM cells, as measured by flow cytometry. **c** Representative confocal images of 5T33MM cells labeled with FITC-conjugated p5 peptide (green), stained with the APC-conjugated anti-mouse IgG antibody (white), and DAPI (blue). The analysis was performed using a Leica TCS SP2 confocal microscope at 40X magnification

The experimental plan undertaken is shown in Fig. 1. Briefly, we screened a *C7C* M13 phage-displayed RPL [*NEB*] using as bait the Igs isolated from cultured 5T33MM cells. The random peptide insert was flanked by a pair of cysteines resulting in cyclized peptide exposed on M13 coat protein. After three rounds of affinity selection, single phage clones were purified and tested by enzyme-linked immunosorbent assay (ELISA) for binding to the 5T33MM Igs or control Igs, as previously described [26]. The insert peptide sequence from selected phage, the percentage of clonal identity, and K_D_ values of identified synthetic Id-peptides are shown in Table 1. Based on the highest affinity binding to 5T33MM Igs, the insert amino acid sequence of phage clone 5 (CIGNSNTLC) was used for large-scale synthesis of the p5 peptide in order to evaluate the binding properties outside of the phage context. To this end, the biotinylated-p5 peptide was incubated with 5T33MM-Igs coated plates at different concentrations, and revealed with streptavidin-conjugated alkaline phosphatase. The p5 peptide showed a concentration-dependent specific reactivity to the cognate antibody, and did not react against an A20 secreted IgG or polyclonal mouse Igs (Fig. 2a).

**Table 1:** Characteristics of 5T33MM Id-peptides

Id-peptide name	Sequence (aa)^a^	Freq. (%)^b^	KD (nM)^c^
p^5^	CIGNSNTLC	38,4	6,29
p^8^	CTVRTSADC	23,2	15.4
p^2^	CSNNGNALC	15,4	47,3
p^4^	CISNGNQPC	15,4	64,2
p^3^	CRVNTAALC	7,6	77,7

^(a)^ Recombinant peptide insert displayed by *C7C* phage-displayed RPL

^(b)^ Percentage of independent clones isolated at the end of three biopanning cycles

^(c)^ K_D_ values for the Id-peptides binding to the cognate mIg-5T33MM, as estimated by Scatchard plot analysis

By flow cytometry, we analyzed the specific binding of FITC-conjugated p5 peptide to the 5T33MM, A20 and IM9 cells expressing the surface Ig-BCR. An irrelevant peptide (CGGNGPGLC) was included as a negative control (pCNT). The p5 peptide recognized 5T33MM target cells in a dose-dependent manner, while the pCNT peptide did not (Fig. 2b). The specific Id-peptide binding to 5T33MM was verified by testing other B-cells, including the IM9 and A20 cell lines and primary B cells from a multiple myeloma patient and healthy donor (Additional file 1: Figure S1). To visualize the binding of the p5 peptide to the surface Igs, we performed confocal microscopy of 5T33MM cells stained with the FITC-conjugated p5 peptide and the anti-mouse IgG detecting the surface Igs. The p5 peptide bound to the 5T33MM cell surface and also localized inside the cells (Fig. 2c). Since this analysis was conducted on focal planes of non-permeabilized cells, the intracellular localization of the p5 peptide was a likely consequence of the BCR-mediated internalization.

**Fig. 3:**
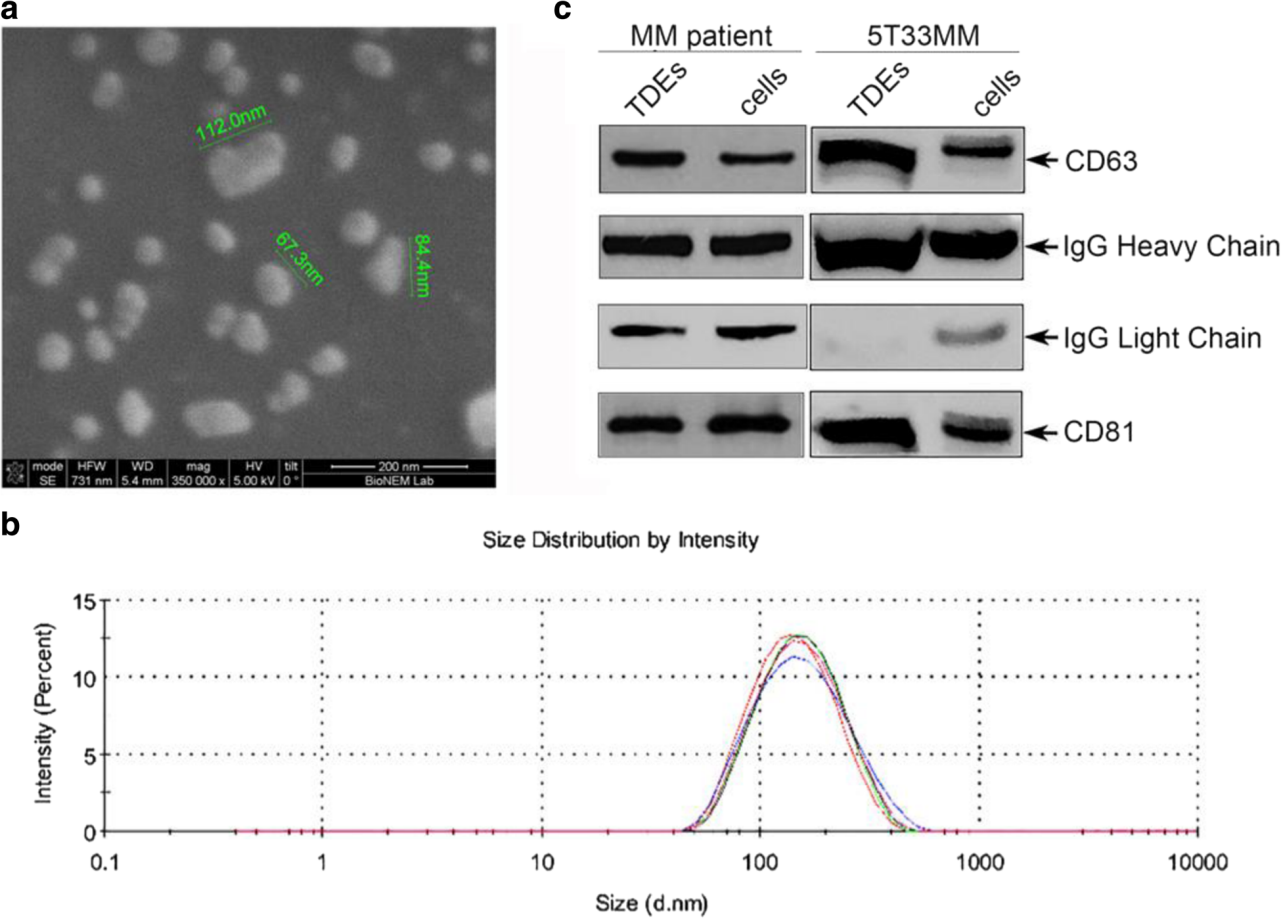
In vitro characterization of exosome preparations. **a** Scanning electron microscopy (SEM). The 5T33MM cell culture supernatant was filtered (0.22 μm) and the exosomes were purified using the ExoQuick-TC™. The SEM analysis was performed at 20, 000 x magnification (SEM FEI Novalab 600). **b** Size distribution of the exosomes population derived from 5T33MM cells using dynamic light scattering (Zetasizer Nano S, Malvern Instruments). **c** B-cells from the blood of multiple myeloma patient and 5T33MM cells as well as purified exosomes from patient serum or 5T33MM cell supernatant were lysed in RIPA buffer, separated by SDS-PAGE, and analyzed by Western blotting using the indicated antibodies

Exosomes were then purified from the supernatant of cultured 5T33MM, A20 and IM9 cells and from serum of MM patient and healthy donor. By scanning electron microscopy (SEM), the exosomes had a rounded shape (30–120 nm diameter) with some agglomerations occurring in the drying process during sample preparation (Fig. 3a). Confirmation of the size homogeneity of vesicles was verified using a Zetasizer Nano ZS90 (Fig. 3b). Purified exosomes were also analyzed by Western blotting using antibodies against typical exosomal markers, such as CD81, CD63, including anti-IgG antibody to verify the IgG expression on TDEs (Fig. 3c).

**Fig. 4:**
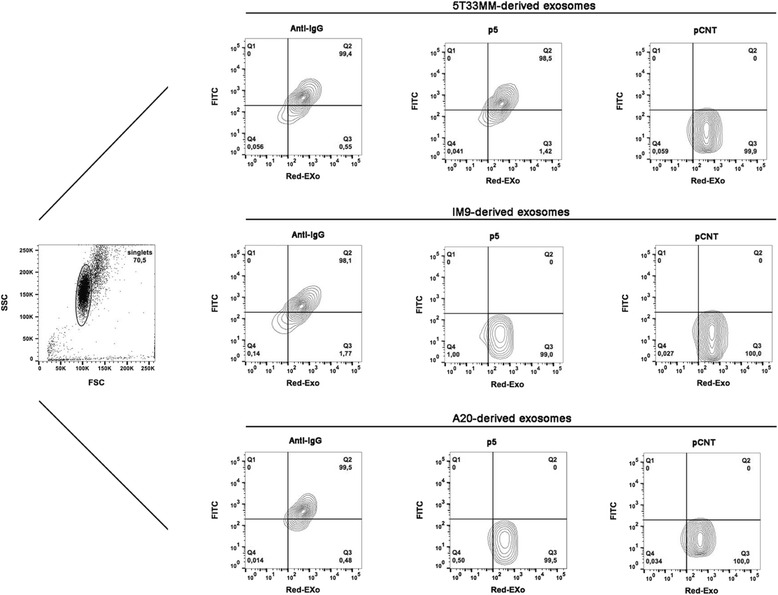
Flow cytometry of purified exosomes derived from cultured B-cells. Exosomes were purified from supernatants of the murine 5T33MM and human IM9 multiple myeloma cells, and the murine A20 B-lymphoma cells. The production of IgG-bearing exosomes was similar in the three cell lines. The p5 peptide recognized specifically the 5T33MM-released exosomes. Front/side scattering of exosome-bound SMNPs (External plot); Exo-Red-stained exosomes incubated with FITC-conjugated anti-IgG (left plots); Exo-Red-stained exosomes incubated with FITC-conjugated p5 peptide (central plots); Exo-Red-stained exosomes incubated with FITC-conjugated pCNT (right plots)

To validate the reliability of the p5 peptide in targeting the 5T33MM-released exosomes, we used an immuno-capture approach based on anti-CD63 decorated streptavidin magnetic nanoparticles (SMNPs) to trap exosomes [27]. The size of the exosomes-functionalized SMNPs (9.1 μm of diameter) supported the use of flow cytometric detection. Front/side scattering (FSC/SSC) plot of RedExo-labeled and un-labeled exosomes is shown in Fig. 4a. 5T33MM–derived exosomes were equally detected using anti-mouse IgG or FITC-conjugated p5 peptide, while undetected when stained with control peptide, or left unstained (Fig. 4b-e). As controls, the FITC-conjugated p5 peptide did not detect the exosomes released from the human IM9 multiple myeloma and murine A20 B-lymphoma cells (Fig. 4). Further, this analysis provided the evidence of similarity between murine and human multiple myeloma derived-exosomes in terms of IgG expression (Fig. 4).

**Fig. 5:**
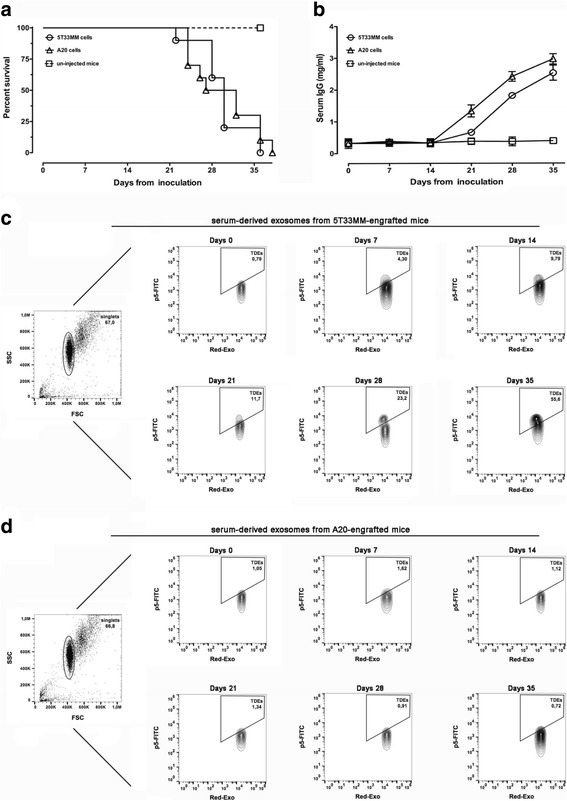
Tumor progression and serum exosomes production in tumor-engrafted mice. **a** Kaplan-Meier survival curves in 5T33MM-engrafted mice (*n* = 10), A20-engrafted mice (n = 10) and un-grafted control mice (n = 10). **b** Determination of serum paraprotein (IgG2b) concentration in 5T33MM- or A20-engrafted mice and control mice measured by ELISA. **c** Flow cytometric analysis of Red-Exo-stained exosomes derived from serum of a representative 5T33MM-engrafted mouse incubated with FITC-conjugated p5 peptide. **d** Flow cytometric analysis of Red-Exo-stained exosomes derived from serum of a representative A20-engrafted mouse incubated with FITC-conjugated p5 peptide

For translating the analysis of MM-released exosomes to in vivo preclinical models, two groups of 10 mice were respectively intravenously inoculated with 5T33MM cells and with A20 cells. A third group of 10 un-injected mice were used as negative control. At day 0 and each 7 days, blood samples were collected by retro-orbital bleeding for measuring the serum MM-released exosomes and paraprotein levels. According to the Kaplan–Meier survival curve the fatal outcome of mice progressively occurred between 21 and 40 days post cells injection (Fig. 5a). By ELISA, the serum paraprotein was detected 21 days after tumor inoculation (Fig. 5b). Exosomes were purified from serum samples, conjugated with SMNPs and analyzed by flow cytometry using the FITC-conjugated p5 peptide. Among 5T33MM serum-derived exosomes, we identified a p5 peptide-positive exosome sub-population 7 days after tumor inoculation, which increased in a time-dependent manner until death (Fig. 5c). Serum-derived exosomes from A20 harboring mice were also purified and used as control (Fig. 5d). The percentage of p5-FITC positive TDEs population in all mice is reported in the Additional file 1: Table S1. These results indicated that analysis of serum MM-released exosomes allowed an earlier detection of MM growth compared to the conventional measure of paraprotein.

## Conclusion

In conclusion, we describe the development of a method allowing the rapid and simple detection of MM-released exosomes using the Id-peptide binders of the Igs expressed by tumor B-cells. Being the IgBCR expressed on the surface of tumor B cells as well as tumor-derived exosomes, our methodology could be extended to the most common B cell neoplasia with potential application for monitoring the minimal residual disease. Furthermore, compared with the standard exosome purification and immuno-capturing systems, the advantage of our method relies on the use of fluorescent-labeled Id-peptides coupled to SMNPs, which represent a more powerful and sensitive tool for tumor detection. Based on specificity, flexibility, cost effectiveness and modularity, our capture system is ideal for novel non-invasive applications in personalized medicine and could be easily extended to other B-cell malignancies. Our experimental approach could open new avenues in devising lab-on-a-chip platforms and liquid biopsy options for an effective early detection of TDEs shed into the blood by tumor B-cells.

## Additional file

Additional file 1: Table S1. Percentage of p5-FITC positive TDEs population after tumor cells injection. **Figure S1.** Flow cytometry analysis of purified exosomes derived from different cell sources (DOC 570 kb)

## Abbreviations

ELISA: Enzyme-linked immunosorbent assay
FITC: Fluorescein isothiocyanate
FSC/SSC: Front/side scattering
Id-peptides: Idiotype peptides
Ig-BCR: B-cell receptor
Igs: Immunoglobulins
mIg: monoclonal immunoglobulin
MM: Multiple myeloma
pCNT: Irrelevant peptide
RPLs: Random peptide libraries
SEM: Scanning electron microscope
SMNPs: Streptavidin magnetic nanoparticles
TDEs: Tumor-derived exosomes
