# Supplementary material for: Monitoring multiple myeloma by idiotype-specific peptide binders of tumor-derived exosomes
Source: Mol Cancer. 2017 Oct 13;16:159. doi: 10.1186/s12943-017-0730-8 (PMC5640902; doi:10.1186/s12943-017-0730-8)
Supplement: Additional file 1: Table S1. — Percentage of p5-FITC positive TDEs population after tumor cells injection. Figure S1. Flow cytometry analysis of purified exosomes derived from different cell sources (DOC 570 kb) [file 12943_2017_730_MOESM1_ESM.doc]

**Additional files**


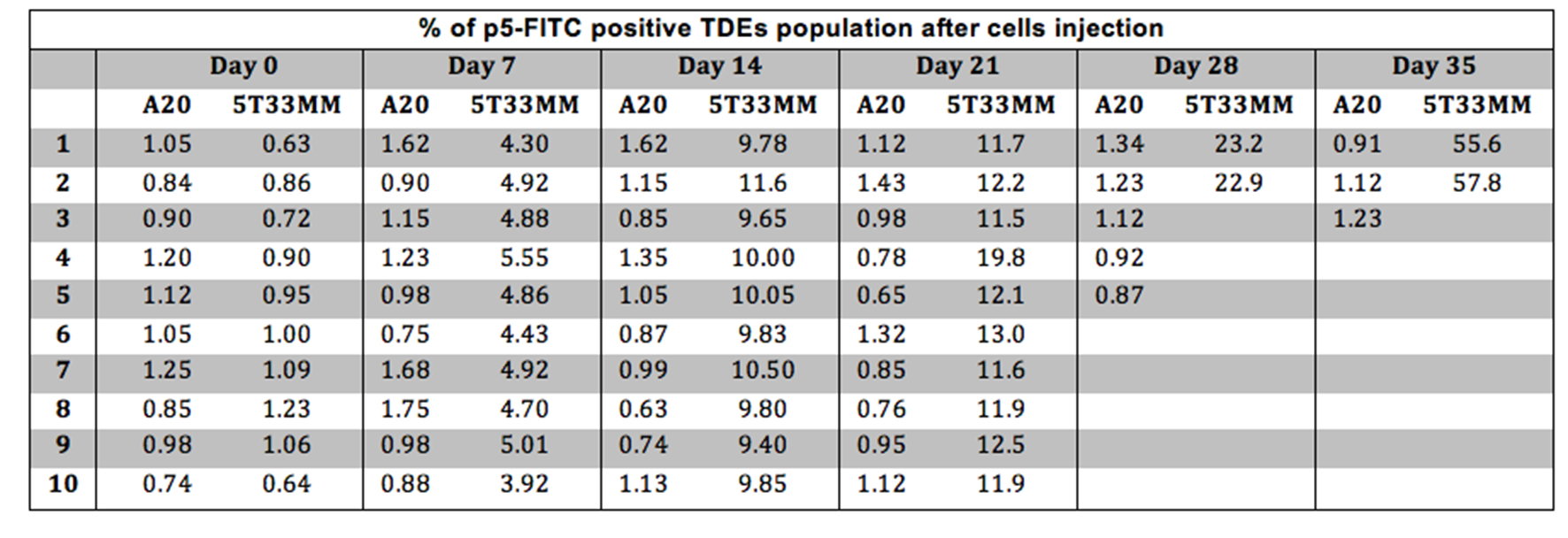


Table S1. Percentage of p5-FITC positive TDEs population after tumor cells injection.


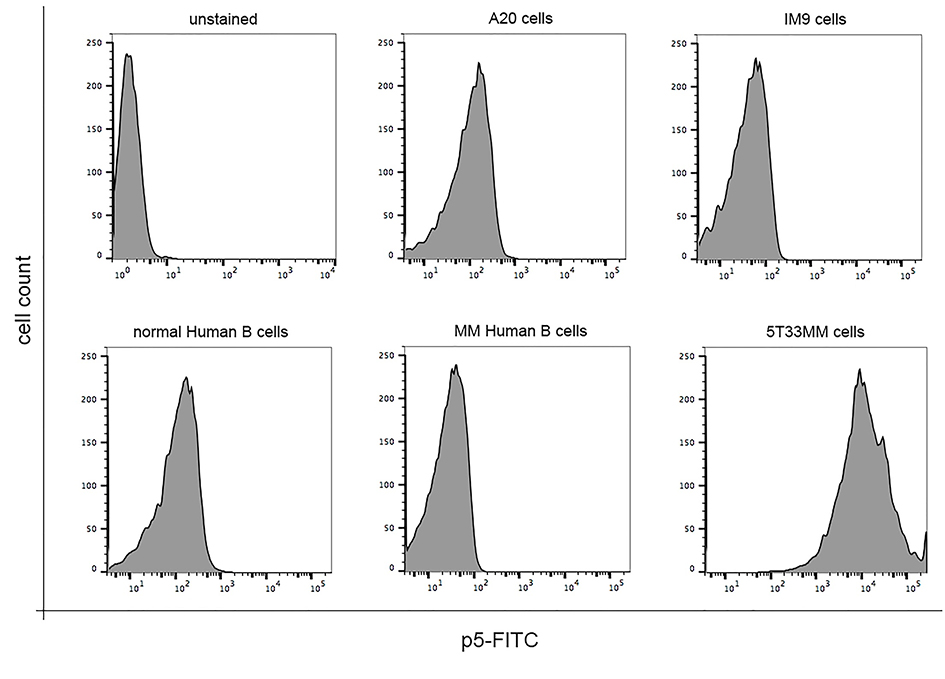


Figure S1. Flow cytometry analysis of purified exosomes derived from different cell sources.
